# A comparison of sample collection methods for quantifying cell-free fetal neurodevelopment transcripts in amniotic fluid

**DOI:** 10.1186/s13104-016-2146-8

**Published:** 2016-07-07

**Authors:** Lisa Hui, Stephen Tong, Tu’Uhevaha J. Kaitu’u-Lino, Natalie J. Hannan

**Affiliations:** Translational Obstetrics Group, Department of Obstetrics and Gynaecology, Mercy Hospital for Women, University of Melbourne, 163 Studley Rd, Heidelberg, VIC 3084 Australia; Public Health Genetics, Murdoch Childrens Research Institute, Parkville, VIC Australia

**Keywords:** Amniotic fluid, Cell-free RNA, Fetal development, qPCR, Gene expression

## Abstract

**Background:**

Cell-free RNA (cfRNA) transcripts known to be expressed by the fetal brain are detectable by quantitative reverse transcription PCR (RT-qPCR) in amniotic fluid and represent potential biomarkers of neurodevelopment. The aim of this study was to compare the cfRNA yields from amniotic fluid (AF) collected in a commercial RNA stabilization product with the traditional method of freezing alone.

**Findings:**

Thirteen women undergoing elective Cesarean birth at term without labor had whole AF collected at the time of uterine incision, prior to membrane rupture. Patient samples were split between Streck RNA blood collection tubes (BCT) and plain sterile polypropylene centrifuge tubes. Cell-free RNA from the AF supernatant was extracted according to a previously published protocol. RT qPCR was performed for the reference gene *GAPDH*, and three genes associated with neurodevelopment (*NRXN3, NTRK3*, and *ZBTB18*). The yield from samples collected in Streck RNA BCT and plain centrifuge tubes were compared with the paired t test. *GAPDH, NRXN3* and *ZBTB18* amplified successfully in all samples, but *NTRK3* did not. The RNA yield was significantly lower in samples collected in the Streck RNA BCT compared with the traditional storage method of freezing alone for all three successfully amplified genes (p < 0.0001).

**Conclusions:**

Selected cfRNA neurodevelopment transcripts are consistently detectable in third trimester AF. There appears to be no benefit in collecting AF in Streck RNA BCT for quantitative studies of AF cell-free RNA.

## Findings

### Introduction

For most couples facing a diagnosis of a fetal abnormality, the projected neurodevelopmental outcome for their baby is of primary importance. To date, our methods of antenatal prediction of long term neurodevelopmental outcome has been confined to only a few modalities—imaging with ultrasound and MRI, and maternal blood analysis for markers of aneuploidy or neural tube defects. A prenatal predictor of neurodevelopmental outcome that is not dependent on the presence of structural brain malformations would be of great value in perinatal medicine.

A novel approach would be to examine the genetic transcripts found in the amniotic fluid that encode genes responsible for neurodevelopment. Amniotic fluid (AF) cell-free RNA (cfRNA) has previously been shown to be a novel source of information about multiple organ systems including the fetal brain [1–3]. The majority of the gene expression studies on AF utilizing cfRNA have been performed with whole genome microarrays, with fewer data from RT-qPCR, which has a greater dynamic range. CfRNA transcripts that are (1) highly expressed by fetal brain, (2) detectable in AF and (3) exhibit brain-specific expression patterns, represent candidate biomarkers of neurodevelopment that could be measured using RT-PCR.

Functional analysis of cfRNA suggests that is more promising than amniocyte RNA as a source of potential biomarkers of fetal neurodevelopment [3]. However, there are technical challenges involved with working with cfRNA, as it can be more degraded that cellular RNA. A prior study has demonstrated that prolonged storage of AF at −80 °C leads to fragmentation of the cell-free nucleic acids [4]. This limits the feasibility of large scale multicentre studies and long term biobanking for AF cfRNA studies.

Commercial RNA stabilization reagents have been developed for use with whole blood (e.g. PAXgene™ blood tubes, Qiagen) and saliva (RNAprotect Saliva™, Qiagen), but not yet for AF. A collection tube for the stabilization of cfRNA in blood (Streck RNA BCT™) is also available. This cfRNA stabilization tube is compatible with the Qiagen Circulating Nucleic Acid kit used for AF RNA extraction, according to the manufacturer.

The aims of this study were (1) to determine if candidate neurodevelopmental transcripts are consistently detectable in AF using RT-qPCR and (2) to compare RNA yields from AF collected in commercial RNA stabilization products with the standard method of freezing alone. The Mercy Health Human Research Ethics Committee approved this study (Ref. #14/20).

### Methods

Consenting participants undergoing undergoing elective Cesarean section without labour were prospectively recruited and had whole AF collected at the time of uterine incision prior to membrane rupture, as previously described [5]. AF samples with visible blood or meconium staining were not included. Amniotic fluid samples were collected intraoperatively and split between Streck RNA BCT and the sterile centrifuge tubes. The aliquots processed according to the standard protocol were centrifuged twice (10 min at 300×*g* and 10 min 1600×*g*) and the supernatant frozen at −80 °C. The whole AF aliquots collected in Streck RNA BCT were kept at room temperature for up to 7 days (as per manufacturer’s instructions) before being centrifuged as above and the supernatant processed for cell-free RNA extraction in the same batch as the paired standard sample.

RNA was extracted with the Qiagen Circulating Nucleic Acid kit according to the previously published method [5]. The volume of AF supernatant for each extraction was 5 ml. The RNA was eluted with 100 μl elution buffer and in-solution DNase digestion was performed as per the manufacturer’s instructions. RNA purification and concentration was then performed with the RNeasy MinElute Clean up and Concentration kit (Qiagen) and the final elution was performed with 16 μl of RNase-free water and stored at −80 °C.

Real-time quantitative PCR (qPCR) was performed after reverse transcription using Applied Biosystems High Capacity cDNA Reverse Transcription Kit according to the manufacturer’s instructions. Quantitative gene expression analyses were performed with inventoried Taqman Gene Expression Assays (Applied Biosystems, Carlsbad, CA, USA) for the reference gene *glyceraldehyde 3*-*phosphate dehydrogenase (GAPDH)*, and three genes associated with neurodevelopment: (1) *neurexin 3(NRXN3),* a neuronal cell surface protein-encoding gene involved in cell recognition and adhesion; (2) *neurotrophic tyrosine kinase receptor type 3 (NTRK3),* cell-surface receptor involved in neurotrophin signaling of cell differentiation; (3) and *zinc finger and BTB domain containing 18* (*ZBTB18, alias ZNF238),* a transcriptional repressor of genes involved in neurodevelopment. These genes were selected on the basis of prior detection in microarray studies of AF [2, 6], documented tissue expression in human fetal brain [7] and fetal brain specific expression (defined as expression >10 multiples of the median in a publicly-available gene expression atlas [8]). The qPCR reactions were performed in duplicate on the CFX 384 (BioRad, Foster City, CA, USA), with the following cycling conditions: 50 °C for 2 min, 95 °C for 10 min, and 40 cycles of 95 °C for 15 s, and 60 °C for 1 min.

### Results

Thirteen women consented to participate and had successful amniotic fluid collection at the time of Cesarean section. All AF samples were processed within 7 days. The reference gene *GAPDH* successfully amplified in all samples. Of the candidate neurodevelopmental genes, *ZBTB18* and *NRXN3* amplified in all samples. However, *NTRK3* was present in very low abundance and failed to amplify in 50 % of total aliquots, precluding any statistical analysis.

**Fig. 1:**
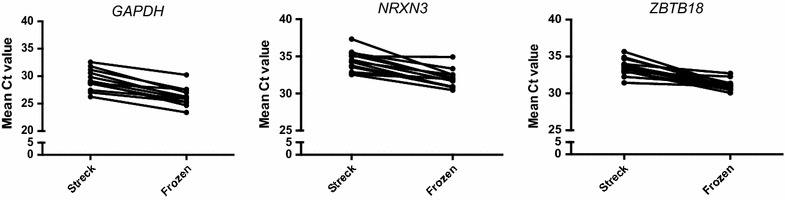
Paired mean Ct values for *GAPDH*, *NRXN3* and *ZBTB18* in amniotic fluid samples stored in Streck RNA BCT at room temperature vs plain centrifuge tubes frozen at −80 °C (p < 0.0001 for all three genes)

For each of the genes, paired t-tests were performed between the mean cycle threshold (Ct) to determine if there was a statistically significant difference in expression according to collection method. The mean Ct results did not demonstrate any improvement in RNA yield with Streck RNA BCT compared with the conventional collection and storage method. In fact, the RNA collected and stored with the conventional method resulted in amplification of all genes at a significantly lower Ct (i.e. higher abundance), with the mean Ct difference of −3.12, −2.39 and −2.47 for *GAPDH*, *NRXN* and *ZBTB18* respectively *(*p < *0.0001* for all three genes*)* (Fig. 1).

### Discussion

This study confirmed that cell-free transcripts *NRXN3* and *ZBTB18* are consistently detectable in term amniotic fluid supernatant. There appears to be no benefit in collecting whole AF in Streck RNA BCT over plain centrifuge tubes for studies of AF cell-free RNA.

One of the major challenges of working with cell-free RNA is assessing the quality of the extracted product. Conventional methods of assessing RNA quality such as the RNA Integrity Number (RIN) produced by the Agilent 2100 Bioanalyzer system uses ribosomal RNA peaks to quantify the quality of RNA. Cell-free RNA does not contain these ribosomal RNA peaks and therefore does not produce a meaningful RIN. In prior work, we found that quantification of *GAPDH* using RT-qPCR correlated well with downstream amplification and microarray hybridization success [9]. In this study, the *GAPDH* yield was lower with the Streck samples compared with the standard methods, indicating no benefit to using Streck BCT for whole AF sample collection.

This lack of improvement with the use of an RNA stabilizing product may be due to the already degraded nature of AF cfRNA at the time of sampling, and implies that there is minimum room for optimization of AF RNA yield with stabilization reagents. Furthermore, the Streck RNA BCT is designed for stabilizing cfRNA in whole blood, and it is not optimized for AF, which may contain additional substances that interfere with its performance.

The failure to consistently detect *NTRK3* in all samples is consistent with prior whole transcriptome analysis demonstrating that neurological system gene expression is relatively downregulated in term samples compared with midgestation [5, 10].

Another potential reason for the low yield was the omission of carrier RNA from the RNA extraction step [10]. This was omitted in the original protocol due to its potential interference in downstream steps such as whole transcriptome amplification. However, it may be suitable to include in future experiments where these considerations are not applicable.

In summary, this study has confirmed the presence of several potential mRNA markers of neurodevelopment with RT qPCR. Further research is required to optimize sample collection and storage for future studies of AF cfRNA.

## Abbreviations

AF: amniotic fluid
BCT: blood collection tube
cfRNA: cell-free RNA
Ct: cycle threshold
GAPDH: glyceraldehyde 3-phosphate dehydrogenase
NRXN3: neurexin 3
NTRK3: neurotrophic tyrosine kinase receptor type 3
RNA: ribonucleic acid
RT qPCR: quantitative reverse transcription polymerase chain reaction
*ZBTB18*: *zinc finger and BTB domain containing* 18 (alias ZNF238)
